# Can Daily Walking Alone Reduce Pneumonia-Related Mortality among Older People?

**DOI:** 10.1038/s41598-020-65440-z

**Published:** 2020-05-22

**Authors:** Takaaki Ikeda, Sumito Inoue, Tsuneo Konta, Masayasu Murakami, Shouichi Fujimoto, Kunitoshi Iseki, Toshiki Moriyama, Kunihiro Yamagata, Kazuhiko Tsuruya, Ichiei Narita, Masahide Kondo, Yugo Shibagaki, Masato Kasahara, Koichi Asahi, Tsuyoshi Watanabe

**Affiliations:** 1 Department of Health Policy Science, Graduate School of Medical Science, Yamagata University, Yamagata, Japan; 2 Department of Cardiology, Pulmonology, and Nephrology, Yamagata University School of Medicine, Yamagata, Japan; 3 Department of Public Health and Hygiene, Yamagata University School of Medicine, Yamagata, Japan; 4 Steering Committee of Research on Design of the Comprehensive Health Care System for Chronic Kidney Disease (CKD) Based on the Individual Risk Assessment by Specific Health Checkup, Fukushima, Japan

**Keywords:** Risk factors, Respiratory signs and symptoms

## Abstract

Pneumonia-related mortality is expected to increase in aging societies. This prospective cohort study examined whether daily walking (1 hour/day) could reduce pneumonia-related mortality among older people who lacked other exercise habits. We analysed data from Japanese Specific Health Checkup across 82 municipalities in 7 prefectures among participants aged ≥65 years who participated in daily walking but did not regularly engage in other forms of exercise (n = 132,448). Information on walking habits and health-related indicators was assessed at a baseline survey conducted between 2008 and 2014. Pneumonia-related and all-cause mortality were followed for a median of 3.4 years. We performed a competing risk model with propensity score matching to evaluate the association between daily walking habits and pneumonia-related mortality. Our propensity-matched analysis revealed a significant association between daily walking and pneumonia-related mortality among older people who lacked other exercise habits, such that the sub-hazard ratio and 95% confidence intervals were 0.58 (0.39, 0.86). This study extends the findings of previous research on the effects of exercise on pneumonia by demonstrating that daily walking alone is sufficient to reduce pneumonia-related mortality among older people who do not regularly engage in other exercise habits.

## Introduction

Lower respiratory tract infection is the fourth leading cause of mortality worldwide. According to the recently published Global Burden of Disease Study, this condition accounted for >1 million deaths in 2017. Its impacts are more severe in ageing societies, where it is the second leading cause of mortality^1^; however, its prevalence is expected to dramatically increase in ageing societies^1^. Indeed, lower respiratory tract infection is currently the third most common cause of mortality in Japan, which has among the world’s highest life expectancy^2^, and it is the leading cause of death of Japanese men^3^. Pneumonia is particularly widespread among lower respiratory diseases impacting older people^4^, and it frequently leads to hospitalisation and mortality^5,6^. Therefore, developing and implementing effective measures to prevent pneumonia in older populations are emerging challenges in ageing societies.

Previous studies have identified the effectiveness of exercise in reducing respiratory-related mortality in both the general population and older age groups^7–10^ For example, two cohort studies from Japan reported that daily walking habits were associated with a lower risk of pneumonia-related mortality^7,8^. However, these investigations did not consider whether participants engaged in other exercise activities. One study examined the association between daily walking habits and pneumonia-related mortality, but the researchers did not concurrently include other exercise habits (playing sports) in the regression models^7^. In another study, the researchers did not consider whether participants engaged in exercise habits other than walking^8^. Moreover, participants analysed in other previous studies were more likely to be younger and healthier than the general older population^9,10^. Thus, it remains unclear whether daily walking alone can reduce pneumonia-related mortality among older people who do not regularly engage in other exercise habits. Herein, we report the results of a large-scale, prospective cohort study in which we explored this possibility in Japan.

## Methods

### Study population and data collection

This study was conducted as part of an ongoing project called ‘Research on the design of a comprehensive health care system for chronic kidney disease’ based on individual risk assessments by the Specific Health Checkup of all Japanese citizens aged 40–74 years^11^. In brief, the Specific Health Checkup is an annual health evaluation of all inhabitants of Japan aged 40–74 years; it is covered by national health insurance and has been ongoing since 2008. Our data were obtained from a prospective cohort study conducted at 82 municipalities in 7 prefectures, in which 664,926 inhabitants (284,320 men and 380,606 women) were enrolled. We identified 132,448 eligible participants after excluding individuals aged ≤64 years (n = 338,160), those who reported other exercise habits during the baseline survey (n = 129,971) and those with missing data on exercise or walking habits (n = 64,347). Information on health-related indicators was assessed using a baseline survey conducted between 2008 and 2014 (Supplementary Fig. 1). Exercise habits other than walking were ascertained based on ‘yes’ and ‘no’ responses to the following question: ‘Do you have the habit of exercising to sweat lightly for >30 min at a time, twice weekly, for over 1 year?’

The following information on health-related behaviours were assessed by a self-reported questionnaire in the baseline survey: age, sex, smoking status (ex-smoker/never smoker or current smoker), alcohol drinking habit (rarely, sometimes or daily), daily walking habits, medication use for hypertension and diabetes mellitus and medical history (previously diagnosed with heart disease/stroke). Daily walking habits were ascertained based on responses of the question ‘In your daily life, do you walk or do any equivalent amount of physical activity for >1 h a day?’ with possible answers of ‘yes’ or ‘no’. Participant’s height, weight and blood pressure were objectively measured by trained staff. Blood pressure was measured using a standard sphygmomanometer or an automated device on the right arm after resting in the sitting position for 5 min. We also obtained laboratory data including fasting plasma glucose and glycated haemoglobin A1c levels. All blood analyses were conducted in local laboratories. Using this subjectively and objectively measured information, we identified participants with diabetes mellitus (fasting plasma glucose level of >126, glycated haemoglobin A1c level of >6.5% or using medication for diabetes mellitus) and/or hypertension (systolic blood pressure level of >140 mm Hg, diastolic blood pressure level of >90 mm Hg or using medication for hypertension) and used these conditions as covariates. We also calculated body mass index (BMI) as weight (in kg) divided by the square of height (in m) and classified BMI into four categories: <18.5, 18.5–24.9, 25.0–29.9 and ≥30. We considered residential municipalities as covariates and coded them as dummy variables.

### Follow-Up

Dates and causes of death were confirmed via death certificates and coded according to the International Classification of Diseases, 10^th^ Revision. Death due to pneumonia (J12–18) was targeted as the primary outcome. The median follow-up period and total follow-up person-years were 3.4 years and 456,286.8 years, respectively.

### Statistical analysis

We used a competing risk model with the Fine and Gray method^12^ using propensity score matching to evaluate the association between daily walking habits and pneumonia-related mortality. To calculate the propensity score of daily walking habits for each participant, we first estimated the propensity score obtained from a logistic regression model using the variables that were included in our competing risk models (age, sex, BMI, smoking status, alcohol drinking habits, medical history of heart disease/stroke, diabetes mellitus, hypertension and residential municipalities). Then, we performed one-to-one and nearest-neighbour matching with a caliper width of one-quarter of the standard deviation of propensity scores without replacement. After matching, we confirmed that the absolute standardised differences (ASDs) between those with and without daily walking habits for all covariates were <0.1. In the competing risk model, missing variables of all covariates were treated as dummy variables.

Regarding sensitivity analysis, the same competing risk models using multiple imputation methods were used. All missing variables were imputed under the missing at random assumption. The variables sex, age, smoking status, alcohol drinking habits, BMI, medical history of heart disease/stroke, hypertension, diabetes mellitus and residential municipalities were imputed by multivariate imputation chained equations^13^. Rubin’s rule was applied to combine results across 10 imputed datasets^13^. We also calculated the E-value to check the robustness of our results against residual confounding factors^14,15^. This value estimates the minimum strength of association between any unmeasured confounder and both daily walking habits and pneumonia-related mortality. Using this method enabled us to additionally examine the effect size of our study along with the sub-hazard ratios (sHRs). We calculated the E-value for the observed sHR using the following formula (1)^14,15^:

1 $${\rm{E}}-{\rm{value}}=1/{\rm{sHR}}+{\rm{sqrt}}[1/{\rm{sHR}}\times (1/{\rm{sHR}}-1)].$$

All analyses were conducted using Stata (version 16.0; Stata Corp, College Station, TX). This study was approved by the Ethics Committee of Yamagata University (Approval No. 2008-103). Data were completely anonymised before being provided to the investigators.

## Results

Table 1 and Supplementary Table 1 summarise the baseline characteristics of the eligible participants (n = 132,448) and matched pairs (n = 44,627). A total of 46,327 participants (35.0%) reported walking for >1 h daily. Among the eligible participants, missing data were observed regarding smoking status (n = 1), alcohol consumption habits (n = 597), medical history of stroke (n = 3,213), medical history of heart disease (n = 3,335), hypertension (n = 71) and diabetes (n = 33,171) (Table 1 and Supplementary Table 1). Men with a BMI of <18.5 who were current smokers and consumed alcohol daily were more likely to die during the follow-up period (Table 2). Among the pre-matching participants, ASDs shown in Table 1 indicated that only residential municipalities identified poorly balanced groups of those who engaged and did not engage in daily walking. After propensity score matching, all ASDs were <0.1, thus indicating that our matching method provided good balance (Table 1 and Supplementary Table 1).

**Table 1:** Baseline characteristics of participants before and after using propensity score matching methods.

	Before matching			After matching		
	No walking habits	Walking habits	ASD	No walking habits	Walking habits	ASD
	n = 86,121	n = 46,327		n = 44,627	n = 44,627	
Sex			0.032			0.014
Women: N (%)	51,883 (60.2)	28,629 (61.8)		27,774 (62.2)	27,465 (61.5)	
BMI, No (%)			0.071			0.020
18.5–24.9	55,169 (64.1)	31,010 (66.9)		30,376 (68.1)	19,902 (67.0)	
<18.5	5,039 (5.9)	2,858 (6.2)		2,614 (5.9)	2,823 (6.3)	
25.0–29.9	22,437 (26.1)	10,949 (23.6)		10,264 (23.0)	10,435 (23.4)	
≥30	3,476 (4.0)	1,510 (3.3)		1,373 (3.1)	1,467 (3.3)	
Smoking status: N (%)			0.052			0.020
Former/Nonsmoker	75,923 (88.2)	41,589 (89.8)		40,265 (90.2)	39,998 (89.6)	
Current smoker	10,197 (11.8)	4,738 (10.2)		4,362 (9.8)	4,629 (10.4)	
Missing	1 (0.0)	—		—	—	
Alcohol habits: N (%)			0.025			0.007
Rarely/Never	50,439 (58.6)	27,634 (59.7)		26,569 (59.5)	26,332 (59.0)	
Sometimes	16,571 (19.2)	9,035 (19.5)		8,644 (19.4)	8,806 (19.7)	
Everyday	18,683 (21.7)	9,489 (20.5)		9,273 (20.8)	9,323 (20.9)	
Missing	428 (0.5)	169 (0.4)		141 (0.3)	166 (0.4)	
Age: mean (SD), years	69.2 (2.9)	69.3 (2.8)	0.018	69.2 (2.9)	69.2 (2.9)	0.001
Residential municipality			**0.102**			0.020

Note: ASD ≥ 0.1 is represented in the bold type. Other variables are presented in Supplementary Table 1. ASD = Absolute standardised difference.

**Table 2:** Differences in the baseline characteristics between participants who survived and died.

	Survived	Died (All cause)	*P* value
	n = 130,117	n = 2,331	
Age: mean (SD), years*	69.2 (2.9)	69.1 (2.6)	0.94
Sex**			
Men: N (%)	50,431 (97.1)	1,505 (2.9)	<0.001
Women: N (%)	79,686 (99.0)	826 (1.0)	
BMI, No (%)**			
18.5–24.9	84,723 (98.3)	31,010 (1.7)	<0.001
<18.5	7,618 (96.5)	2,858 (3.5)	
25.0–29.9	32,872 (98.5)	10,949 (1.5)	
≥30	4,904 (98.4)	82 (1.6)	
Smoking status: N (%)**			
Former/Nonsmoker	115,706 (98.5)	1,806 (1.5)	<0.001
Current smoker	14,410 (96.5)	525 (3.5)	
Alcohol habits: N (%)**			
Rarely/Never	76,817 (98.4)	1,256 (1.6)	<0.001
Sometimes	25,195 (98.4)	411 (1.6)	
Everyday	27,516 (97.7)	656 (2.3)	

^*^ t-test was performed. **Chi-squared test was performed.

Among the 132,448 participants (before matching procedures), 2,331 died during the study period including 87 participants who died of pneumonia (Supplementary Table 2). Among the matched participants (n = 89,254), 58 died of pneumonia and 1,443 of other causes. The sHRs of pneumonia-related mortality according to daily walking habits are shown in Table 3. Participation in daily walking was negatively associated with pneumonia-related mortality after adjusting for covariates: sHR [95% confidence interval (95% CI)] were 0.58 (0.39, 0.86) in the propensity-matched sample. A similar result was observed when using the same competing risk model for multiply imputed datasets: sHR (95% CI) was 0.67 (0.46, 0.97).

**Table 3:** Daily walking habits and pneumonia-related mortality.

	PS matching method			Multiply imputed data set		
	sHR	95% CI		sHR	95% CI	
No walking habits	1.00			1.00		
Walking habits	0.58	0.39	0.86	0.67	0.46	0.97

Note: All models were adjusted for age, sex, body mass index (BMI), smoking status, alcohol drinking habits, past history of heart diseases and stroke, hypertension, diabetes mellitus and residential municipalities. PS = propensity score; sHR = sub-hazard ratio.

The E-value of the point estimate based on our main result [sHR (95% CI) 0.58 (0.39, 0.86)] was 2.84 (lower and upper confidence interval limits: 1.60 and 4.57, respectively). This result indicates that the observed OR of 0.58 could be explained by an unmeasured confounder that was associated with both daily walking habits and pneumonia-related mortality by an sHR of 2.84 each, over and above the measured confounders.

## Discussion

We examined whether daily walking habit could reduce pneumonia-related mortality among older people aged ≥65 years in Japan who did not regularly engage in other forms of exercise and found that walking for >1 h daily was inversely associated with pneumonia-related mortality in this population.

To the best of our knowledge, our study is the first to demonstrate the preventive effects of daily walking habits on pneumonia-related mortality among older people who did not regularly engage in other forms of exercise. Previous research has showed the effects of walking on adverse health-related outcomes such as disability and all-cause mortality^10,16,17^. Our findings are consistent with those of recent studies that indicated that walking has a protective effect on pneumonia-related mortality among both general and older populations^7–10^. Additionally, two cohort studies in Japan found a significant correlation between daily walking for >1 h daily and reduced pneumonia-related mortality among participants aged ≥40 years (mean age of 58 years old) as well as older people without a history of myocardial infarction or stroke (mean age of 71 years old)^7,8^. Other cohort studies have associated walking habits with reduced cardiovascular and pneumonia-related mortality among older participants with comorbid conditions who were at a higher risk of adverse health outcomes^8,18^. To complement and extend these findings, we have added evidence that walking for >1 h daily can reduce pneumonia-related mortality even among older people who lack other exercise habits. However, we could not assess physical fitness deficits such as sarcopenia and frailty, leaving room for debate that a daily walking habit is a proxy for physical fitness^19^. Thus, future studies are needed to determine whether daily walking habits are beneficial for preventing pneumonia-related mortality among older people with a lower level of physical fitness.

The physical activity guidelines, in general, recommend 30 min of daily aerobic activities^20,21^, a shorter duration than that used in our study. Moreover, a cohort study from Japan reported that regular walking for >1 h daily was associated with a reduction in pneumonia-related mortality among older people compared with 30 min daily; regular walking for <30 min daily was associated with an increase in pneumonia-related mortality in this population compared with 30 min daily^8^. Although previous studies have shown that time spent walking decreases with age^22,23^, there is a possibility that maintaining 30-min walking daily can prevent pneumonia-related mortality. Thus, future longitudinal studies that repeatedly assess health-related indicators are expected to clarify the cut-off of the length of time spent walking which is beneficial for reducing pneumonia-related mortality.

A possible pathway can be identified for the inverse association between daily walking habits and pneumonia-related mortality. According to previous research, exercise can improve immune function^24–29^, and several researchers have reported that walking can enhance immune function in older populations. For example, one study revealed that 30-min walking for 5 days a week significantly enhanced the mucosal immune system, as evaluated by higher salivary secretory immunoglobulin A levels (but did not improve level lymphocytes)^28^. Similarly, Shimizu et al. reported significantly higher salivary secretory immunoglobulin A levels among older people who walked 7,000 steps daily than those who walked 3,000 steps daily^29^. Thus, although the immune system generally becomes attenuated with age^30^, daily walking might enhance its ability to defend against pathogens that cause pneumonia and pneumonia-related mortality. However, future studies are expected to address this issue because we did not assess relevant parameters regarding immune function.

Our findings have several important implications. Sedentary lifestyles, which have become increasingly prevalent worldwide, are associated with a range of adverse health outcomes including obesity and all-cause mortality^31–35^. Approximately 60% of older people reported sitting for >4 h daily^36^, thus putting this age group at a particularly high risk for both conditions. American public health guidelines recommend people to be engage in 150- or 75-min moderate or vigorous aerobic physical activity, respectively, per week^20^. Walking is a physical activity which confers various health benefits and is common among older people. Moreover, significantly reduced monthly medical costs have been associated with walking for >1 h daily compared with walking for >30 min daily among community dwellers aged >40 years^37^. Because pneumonia is one of the leading causes of hospitalisation, particularly among older people^6^, such findings indicate that promoting walking habits is effective not only for improving health but also for reducing medical care expenditures.

A major strength of this study was the inclusion of >600,000 participants; this large sample size was sufficient to detect an association between daily walking habits and pneumonia-related mortality. However, several limitations should be noted. First, we could not fully consider confounders such as socioeconomic status (SES), although we adjusted for this influence by including BMI and smoking status—which have previously been associated with SES^38–40^—in our regression models. Moreover, we could not identify another comorbidity status (e.g. depression^41^, chronic obstructive pulmonary disease^42^, and lung cancer^43^). Nevertheless, the E-value of 2.78 can be considered large enough for the effect of daily walking habits when referring to previous reports on the effect of SES on all-cause mortality^41–45^. Thus, we believe that our results are robust even when considering residual confounding factors. Second, we could not assess participants’ cognitive function; thus, the accuracy of the survey responses of some participants with cognitive decline might be limited. However, we analysed participants aged 65–75 years in whom the prevalence and incidence of dementia are relatively low^46^. Third, the participants’ types of walking habits (accumulated or continuous), walking circumstances (walking for pleasure/exercise or walking as part of daily life tasks) and frequency of walking habits were also unconsidered in the present study. Fourth, we could not consider variations in exposure over time because we assessed walking habits using the baseline survey. It is possible that some participants’ daily walking or other exercise habits changed over the course of the study period, and our results might have been overestimated or underestimated. However, two previous observational studies reported that time spent walking among older people decreased during the respective study periods^22,23^. These studies indicate that the walking habits of our participants most likely decreased during the study period. Thus, we believe that assessing health-related indicators only at the baseline is moderately enough to examine the association between walking habits and pneumonia-related mortality. Fifth, we used a self-reported questionnaire to assess daily walking habits, leading to the possibility of residual reporting bias. Moreover, a Japanese observational study using objectively measured physical activity data in older people reported the importance of considering accumulated physical activities evaluated based on physical activity guidelines—including not only moderate-intensity activities but also low-intensity and short-term activities such as chores^47^. In addition, recent research has pointed out the beneficial effect of accumulated physical activity on health outcomes^48^. Thus, future studies using objectively measured walking habits are also needed. Sixth, the generalizability of our study results to other countries and regions remains unclear because our participants were not nationally representative but rather enrolled from 82 municipalities in 7 of 47 prefectures of Japan. Moreover, only 3.7% of the total number of deaths were pneumonia related, much lower than the average number in Japan, because we analysed participants aged 65–75 years, who were relatively younger than people in high-risk populations^49^.

## Conclusion

A significant association was found between daily walking habits and pneumonia-related mortality among older people who did not engage in other forms of exercise. Our findings suggest that enhancing walking activity among people with sedentary lifestyle is an important strategy to tackle pneumonia and pneumonia-related mortality in older populations.

## Supplementary information

Supplementary information.

## Data Availability

The dataset used in this study is not publicly available due to a restriction by agreement among the research group members.
