# Supplementary material for: Can Daily Walking Alone Reduce Pneumonia-Related Mortality among Older People?
Source: Sci Rep. 2020 May 22;10:8556. doi: 10.1038/s41598-020-65440-z (PMC7244731; doi:10.1038/s41598-020-65440-z)
Supplement: Supplementary file 1 — Supplementary information. [file 41598_2020_65440_MOESM1_ESM.docx]

**Title: Can Daily Walking Alone Reduce Pneumonia-Related Mortality among Older People?**

Author names:

Takaaki Ikeda^1^, Sumito Inoue^2^, Tsuneo Konta^3,4^, Masayasu Murakami^1^, Shouichi Fujimoto^4^, Kunitoshi Iseki^4^, Toshiki Moriyama^4^, Kunihiro Yamagata^4^, Kazuhiko Tsuruya^4^, Ichiei Narita^4^, Masahide Kondo^4^, Yugo Shibagaki^4^, Masato Kasahara^4^, Koichi Asahi^4^, Tsuyoshi Watanabe^4^.

Affiliations:

1. Department of Health Policy Science, Graduate School of Medical Science, Yamagata University, Yamagata, Japan.
2. Department of Cardiology, Pulmonology, and Nephrology, Yamagata University School of Medicine, Yamagata, Japan
3. Department of Public Health and Hygiene, Yamagata University School of Medicine, Yamagata, Japan
4. Steering Committee of Research on Design of the Comprehensive Health Care System for Chronic Kidney Disease (CKD) Based on the Individual Risk Assessment by Specific Health Checkup. Fukushima, Japan.


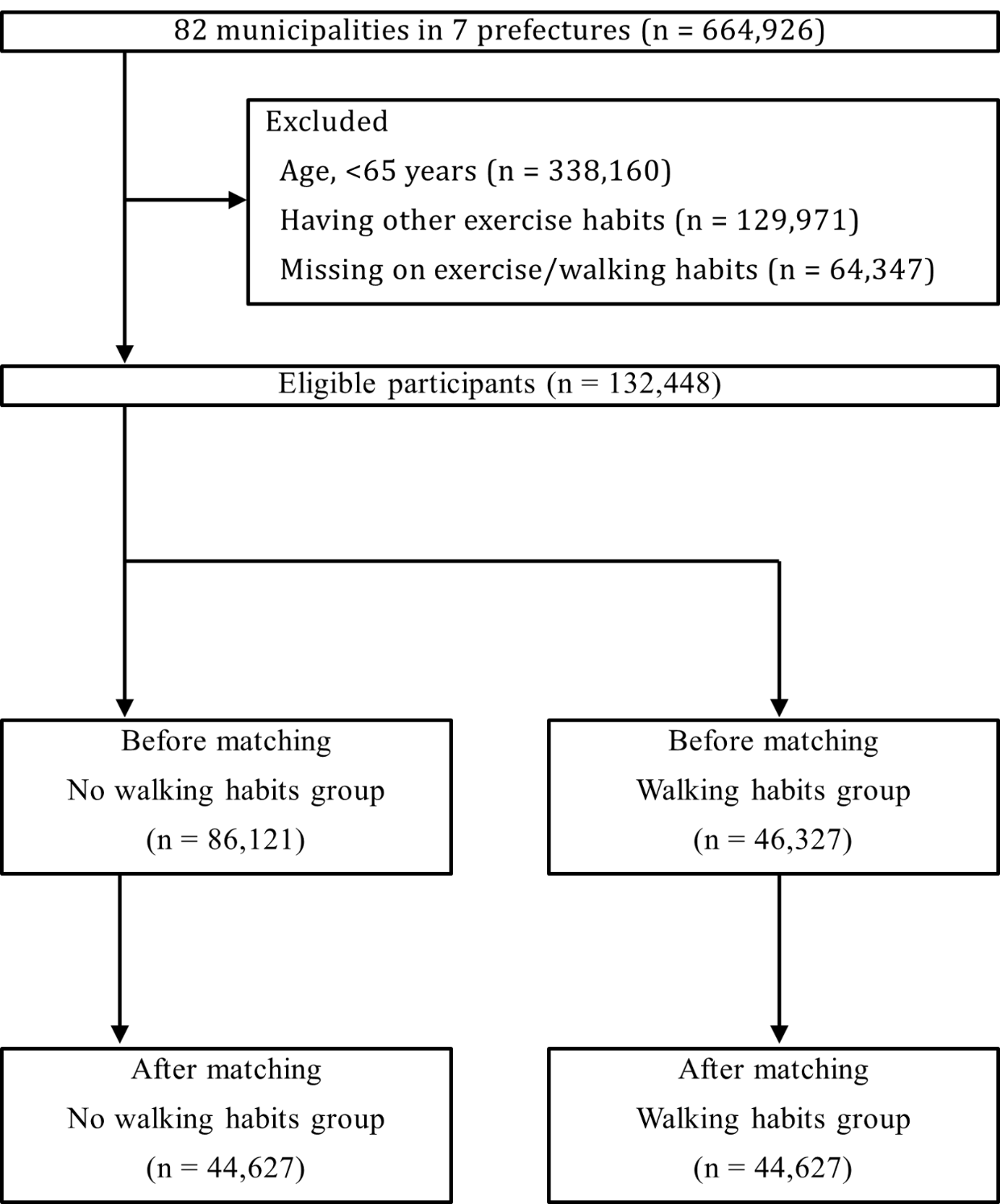


Supplementary Figure 1. Follow-up diagram

Supplementary Table 1. Baseline characteristics of participants before and after using propensity score matching methods

|  | Before matching | | | After matching | | |
| --- | --- | --- | --- | --- | --- | --- |
|  | No walking habits | Walking habits | ASD | No walking habits | Walking habits | ASD |
|  | n = 86,121 | n = 46,327 |  | n = 44,627 | n = 44,627 |  |
| Sex |  |  | 0.032 |  |  | 0.014 |
| Women: N (%) | 51,883 (60.2) | 28,629 (61.8) |  | 27,774 (62.2) | 27,465 (61.5) |  |
| BMI, No (%) |  |  | 0.071 |  |  | 0.020 |
| 18.5–24.9 | 55,169 (64.1) | 31,010 (66.9) |  | 30,376 (68.1) | 19,902 (67.0) |  |
| <18.5 | 5,039 (5.9) | 2,858 (6.2) |  | 2,614 (5.9) | 2,823 (6.3) |  |
| 25.0–29.9 | 22,437 (26.1) | 10,949 (23.6) |  | 10,264 (23.0) | 10,435 (23.4) |  |
| ≥30 | 3,476 (4.0) | 1,510 (3.3) |  | 1,373 (3.1) | 1,467 (3.3) |  |
| Smoking status: N (%) |  |  | 0.052 |  |  | 0.020 |
| Former/Nonsmoker | 75,923 (88.2) | 41,589 (89.8) |  | 40,265 (90.2) | 39,998 (89.6) |  |
| Current smoker | 10,197 (11.8) | 4,738 (10.2) |  | 4,362 (9.8) | 4,629 (10.4) |  |
| Missing | 1 (0.0) | - |  | - | - |  |
| Alcohol habits: N (%) |  |  | 0.025 |  |  | 0.007 |
| Rarely/Never | 50,439 (58.6) | 27,634 (59.7) |  | 26,569 (59.5) | 26,332 (59.0) |  |
| Sometimes | 16,571 (19.2) | 9,035 (19.5) |  | 8,644 (19.4) | 8,806 (19.7) |  |
| Everyday | 18,683 (21.7) | 9,489 (20.5) |  | 9,273 (20.8) | 9,323 (20.9) |  |
| Missing | 428 (0.5) | 169 (0.4) |  | 141 (0.3) | 166 (0.4) |  |
| Age: mean (SD), years | 69.2 (2.9) | 69.3 (2.8) | 0.018 | 69.2 (2.9) | 69.2 (2.9) | 0.001 |
| Past history of stroke: N (%) |  |  | 0.049 |  |  | 0.007 |
| No | 78,795 (91.5) | 43,075 (93.0) |  | 41,405 (92.8) | 41,405 (92.8) |  |
| Yes | 5,136 (6.0) | 2,229 (4.8) |  | 2,204 (4.9) | 2,204 (5.0) |  |
| Missing | 2,190 (2.5) | 1,023 (2.2) |  | 1,018 (2.3) | 1,018 (2.3) |  |
| Past heart disease history: N (%) |  |  | 0.028 |  |  | 0.018 |
| No | 76,708 (89.1) | 41,642 (89.9) |  | 40,018 (89.7) | 40,018 (89.7) |  |
| Yes | 7,151 (8.3) | 3,612 (7.8) |  | 3,541 (7.9) | 3,541 (7.9) |  |
| Missing | 2,262 (2.6) | 1,073 (2.3) |  | 1,068 (2.4) | 1,068 (2.4) |  |
| Hypertension: N (%) |  |  | 0.003 |  |  | 0.013 |
| No | 57,484 (66.8) | 30,991 (66.9) |  | 29,903 (67.0) | 29,903 (67.0) |  |
| Yes | 28,587 (33.2) | 15,315 (33.1) |  | 14,714 (33.0) | 14,714 (33.0) |  |
| Missing | 50 (0.1) | 21 (0.1) |  | 10 (0.0) | 10 (0.0) |  |
| Diabetes: N (%) |  |  | 0.018 |  |  | 0.000 |
| No | 55,343 (64.3) | 30,427 (65.7) |  | 29,205 (65.4) | 29,205 (65.4) |  |
| Yes | 9,165 (10.6) | 4,342 (9.4) |  | 4,192 (9.4) | 4,192 (9.4) |  |
| Missing | 21,613 (25.1) | 11,558 (25.0) |  | 11,230 (25.2) | 11,230 (25.2) |  |
| Residential municipality |  |  | **0.102** |  |  | 0.020 |

Note: ASD ≥0.1 is represented in the bold type. Because of rounding, percentages do not add up exactly to 100%. ASD = Absolute standardised difference

Supplementary Table 2. Reason for death in the eligible participants

| Cause of death | N (%) | ICD–10 |
| --- | --- | --- |
| Injury, poisoning and certain other consequences of external causes | 192 (8.2) | S00–T98 |
| Diseases of the circulatory system | 468 (20.1) | I00–I99 |
| Congenital malformations, deformations and chromosomal abnormalities | 3 (0.1) | Q00–Q99 |
| Endocrine, nutritional and metabolic diseases | 23 (1.0) | E00–E90 |
| Diseases of the digestive system | 68 (2.9) | K00–K93 |
| Diseases of the genitourinary system | 13 (0.6) | N00–N99 |
| Diseases of the blood and blood-forming organs and certain disorders involving the immune mechanism | 9 (0.4) | D50–D89 |
| Certain infectious and parasitic diseases | 43 (1.8) | A00–B99 |
| Symptoms, signs and abnormal clinical and laboratory findings, not elsewhere classified | 39 (1.7) | R00–R99 |
| Diseases of the musculoskeletal system and connective tissue | 15 (0.6) | M00–M99 |
| Neoplasms | 1,225 (52.6) | C00–D48 |
| Diseases of the nervous system | 34 (1.5) | G00–G99 |
| Mental and behavioral disorders | 5 (0.2) | F00–F99 |
| Pneumonia | 87 (3.7) | J12–18 |
| Diseases of the respiratory system other than pneumonia | 102 (4.4) | J00–J11, J19–J99 |
| Unknown | 5 (0.2) | - |

ICD–10, International Classification of Diseases, 10th Revision.
